# News and Notes

**Published:** 1901-12

**Authors:** 


### *NEWS AND NOTES.*

---

THE *Atlanta Medical and Surgical Journal* and *Southern Medical Record* having been consolidated into the ATLANTA JOURNAL-RECORD OF MEDICINE, exchanges will please be sent only to the latter. We are getting many duplicates.

---

RARE CHANCE—DRUG STORE FOR SALE in town of 3,000 inhabitants. Only drug store in town. Sales average $12 to $15 a day, but can be greatly increased by a competent pharmacist. Invoices about $3,000. Owner, a practicing physician, cannot give it his attention. Address J. M., care this Journal.

---

A $60,000 ADDITION will be built on St. Luke's Hospital, Denver.

---

A MEDICAL SOCIETY has been formed by the physicians of Manila.

---

THE open-air treatment of tuberculosis has proved most effective in Germany.

---

SURGEON-GENERAL W. TAYLOR has been appointed Director-General of the British Army Medical Service.

---

THE attendance at the University of Texas, medical department, has increased fifty per cent. over last year.

---

RECENT reports from Alaska show that the natives have suffered a fearful loss of lives from last year's epidemic of smallpox.

THE dental course of the University of Pennsylvania, beginning with the session of 1902–3, will be increased from three to four years.

---

THE street lamp-posts of Paris will be equipped with emergency boxes containing dressings for wounds and an alarm with which to summon an ambulance.

---

A BILL which has been passed by the Georgia House makes it a penal offense to import into that State cattle suffering from any disease except distemper.

---

THE Alaskan quarantine regulations which were enforced against that territory last May have been raised, smallpox having disappeared at all points along the Alaskan coast.

---

DR. HOWARD A. KELLY, of Baltimore, has 150 editions of Hippocrates' works. This is considered the finest and most complete collection of his writings in America, if not in the world.

---

TEXAS fever prevails to such an extent among the cattle in Texas that the Governor of New Mexico has issued a proclamation for quarantine against Texas cattle from November 1 to March 5.

---

UNIVERSITY COLLEGE OF MEDICINE, Richmond, Va., authorities have passed a new law providing that in future credit of one year in the medical course will not be allowed merely because a student has an academic degree.

---

A BARBERS' bill modeled after the Minnesota law on the subject and providing for the proper examination and issuance of certificates to journeymen barbers and the sanitation of barber shops has been introduced into the Georgia House.

THE Memoirs and Letters of Sir James Paget, edited by his son, Mr. Stephen Paget, have just been published in London. The work, both from the high importance of the subject and the competence of its editor, is one of the most valuable and instructive contributions on medical biography ever published.

---

THE Tennessee Board of Pharmacy have found adulteration of drugs in their own State, and have evidence of its prevailing elsewhere. Four offending Nashville druggists whose wares were found adulterated with 50 per cent. of water or other ingredients have been indicted, and other indictments are anticipated. The State law requires that drugs must be 100 per cent. pure, the standard of the United States Pharmacopeia.

---

THE McDowell Building in Danville, Ky., where Dr. Ephraim McDowell performed the first successful ovariotomy, was sold at auction October 21, in order to effect a division of property interests of the owners. The Central Kentucky Medical Association had made unsuccessful efforts in the past to purchase the structure as a meeting place for the organization. Owing to the growth of the town the building is in an undesirable location.

---

DR. GARNAULT, the French physician, has, according to *American Medicine*, arrived in Berlin, and offered himself to Professor Koch for experiments on the identity of human tuberculosis. Professor Koch declined to give injections, but advised him to drink unsterilized milk from a tuberculous cow for one year, and as far as possible abstain from other foods. This he will do, but he will have injections of highly virulent bacilli every month.

---

GOVERNMENT HOSPITAL FOR THE INSANE.—The work on the construction of 12 buildings of the extension located within the main asylum grounds, will be pushed forward. There was a failure in the negotiations to effect an exchange of land between the owners of Wilson Park, Congress Heights, and the Department of the Interior, which were carried on under the authority of Congress, with the aim of securing a better site for the extension buildings.

---

THE following physicians were matriculated at the New Orleans Polyclinic the early part of November. From Texas: Drs. D. D. Hamilton, G. M. Jones, J. F. Scruggs, J. W. Vermillion, A. B. Kennedy, L. Smith, A. M. Horner. From Oklahoma Territory: Dr. R. D. Lowther. From Arkansas: Dr. J. E. Little. From Mississippi: Dr. C. E. Bernham. From Virginia: Dr. R. H. Davis. From Louisiana: Dr. Jas. P. Parker. From Indiana: Dr. Vesta M. Swarts.

---

VIRCHOW'S eightieth birthday was celebrated in Berlin on October 12. Prominent men of science from all parts of the world were assembled for the occasion, as well as distinguished officials, the entire Berlin faculty of medicine, and deputations from all the German universities. Professor Virchow received an ovation at the Pathologic Institute, and made a speech on the "Development of Pathologic Science," which lasted nearly two hours. In the evening there was a banquet given in the lobby of the Lower House of the Prussian Diet, lasting until midnight. Professor Waldeyer, secretary of the Academy of Science, presented Professor Virchow with 50,000 marks, subscribed by the medical men of Germany, to increase the endowment of the Virchow Institute. Emperor William conferred upon him the Great Gold Medal for Science, and the King of Italy sent a gold medallion bearing a portrait of himself. In New York, in celebration of the occasion, one hundred eminent physicians gathered at a banquet presided over by Prof. William Osler, of Johns-Hopkins University, and in Milwaukee it was similarly observed.

---

THE committee on the Senn medal beg leave to call attention to the following conditions governing the competition for this medal for 1902: (1) A gold medal of suitable design is to be conferred upon the member of the American Medical Association who shall present the best essay upon some surgical subject. (2) This medal will be known as the Nicholas Senn Prize Medal. (3) The award will be made under the following conditions: (*a*) The name of the author of each competing essay shall be enclosed in a sealed envelope bearing a suitable motto or device, the essay itself bearing the same motto on device. The title of the successful essay and the motto or device are to be read at the meeting at which the award is made, and the corresponding envelope is to be then and there opened, and the name of the successful author annuunced. (*b*) All successful essays become the property of the Association. (*c*) The medal shall be conferred and honorable mention made of the two other essays considered worthy of this distinction, at a general meeting of the Association. (*d*) The competition is to be confined to those who, at the time of entering the competition, as well as at the time of conferring the medal, shall be members of the American Medical Association. (*e*) The competition for the medal will be closed three months before the next annual meeting of the American Medical Association, and no essays will be received after March 1, 1902. Communications may be addressed to any member of the committee, consisting of the following: Dr. Herbert L. Burrell, 22 Newbury Street, Boston, Mass.; Dr. Edward Martin, 415 South Fifteenth Street, Philadelphia, Pa.; Dr. Charles H. Mayo, Rochester, Minn.

AT the meeting of the Philadelphia County Medical Society, November 13, Dr. Jay F. Schamberg read for himself and Dr. William Welch a paper on vaccination. The course of a typical vaccine pock was described before considering the question of what constitutes a successful revaccination, as it is no more reasonable to expect a typical course in a revaccination than to expect genuine smallpox to follow after vaccination. A successful primary vaccination should be expected to modify a secondary one. The modified course of a revaccination, however, removes all liability to smallpox. A common false vaccine condition is that in which at the end of about seven days there is a hard, reddened area resembling a nevus, but no vesicle. This is not protective. The condemnation of glycerinized vaccine lymph by some physicians was discussed. At the Municipal Hospital it is used to the exclusion of all other virus, and is giving entire satisfaction in both primary and secondary vaccinations. The various results by different observers may be due to two reasons: (1) The material from different manufacturers may not be equally reliable, or (2) there is a difference in the observer's standards as to what constitutes true vaccination. If the first is the reason, government control of all virus is suggested. Tightly-fitting shields over vaccination points are condemned. They are useful for a few hours, while the serum is drying, but their continuous use does more harm than good.

